# Synergistic Anti-*Candida* Activity of Bengazole A in the Presence of Bengamide A †

**DOI:** 10.3390/md17020102

**Published:** 2019-02-07

**Authors:** Matthew T. Jamison, Xiao Wang, Tina Cheng, Tadeusz F. Molinski

**Affiliations:** 1 Department of Chemistry, University of California, San Diego, 9500 Gilman Drive MC0358, La Jolla, CA 92093, USA; mx.jamison@gmail.com (M.T.J.), xiao.wang1@merck.com (X.W.), tina.cheng@sirenasmd.com (T.C.); 2 Skaggs School of Pharmacy and Pharmaceutical Sciences, University of California, San Diego, 9500 Gilman Drive MC0358, La Jolla, CA 92093, USA

**Keywords:** antifungal, alkaloid, synergism, *Candida albicans*

## Abstract

Bengazoles A–G from the marine sponge *Jaspis* sp. exhibit potent in vitro antifungal activity against *Candida* spp. and other pathogenic fungi. The mechanism of action (MOA) of bengazole A was explored in *Candida albicans* under both liquid culture and surface culture on Mueller-Hinton agar. Pronounced dose-dependent synergistic antifungal activity was observed with bengazole A in the presence of bengamide A, which is also a natural product from *Jaspis* sp. The MOA of bengazole A was further explored by monitoring the sterol composition of *C. albicans* in the presence of sub-lethal concentrations of bengazole A. The GCMS of solvent extracts prepared from liquid cultures of *C. albicans* in the presence of clotrimazole―a clinically approved azole antifungal drug that suppresses ergosterol biosynthesis by the inhibition of 14α-demethylase―showed reduced cellular ergosterol content and increased concentrations of lanosterol and 24-methylenedihydrolanosterol (a shunt metabolite of ergosterol biosynthesis). No change in relative sterol composition was observed when *C. albicans* was cultured with bengazole A. These results eliminate an azole-like MOA for the bengazoles, and suggest that another as-yet unidentified mechanism is operative.

## 1. Introduction

**Figure 1:**
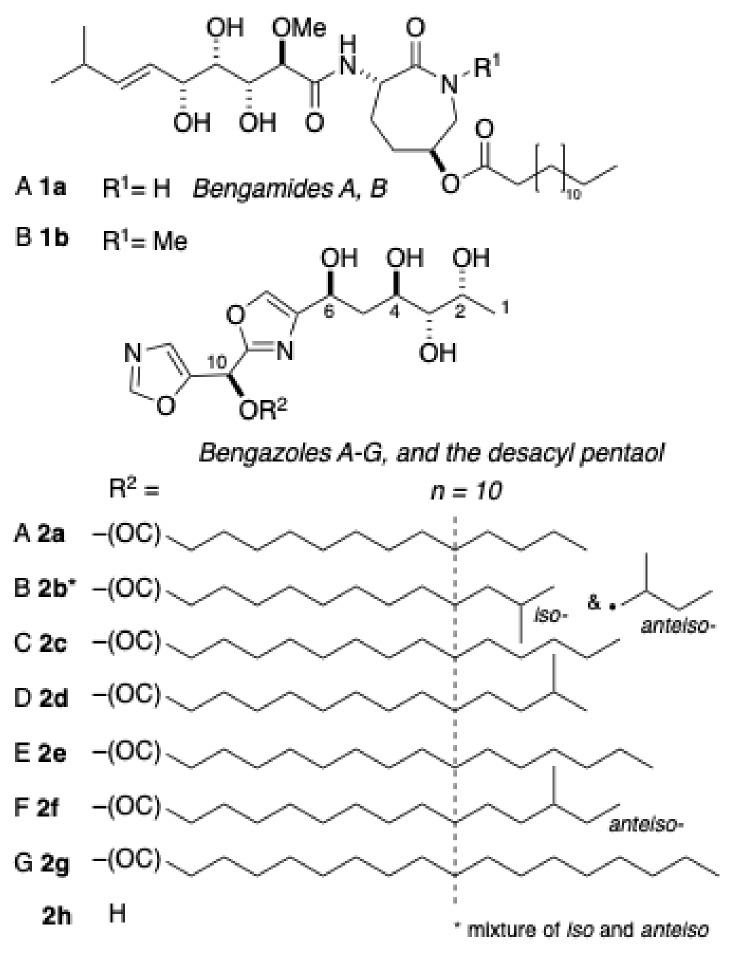
Structures of bengamides and bengazoles.

The tropical sponge, *Jaspis* cf. *coriacea*, is the original source of two classes of unrelated natural products: bengazoles and bengamides, (Figure 1) first reported by Crews et al. [1]. Bengamides A (**1a**) [2,3] and B (**1b**) and related analogs are potent nanomolar inhibitors of cancer cell growth, with selective in vitro activity in the NCI 60 cell line panel [4]. Compound **1a** reduced MDA-MB-435 breast carcinoma in an in vivo rat zenograft model [4]. The unique cytotoxicity of bengamides has been attributed to the inhibition of methionine aminopeptidases [5,6]. A synthetic analog of **1a**, LAF-389 [7], was advanced to phase I clinical trials before discontinuation of the study due to intolerable toxicity [8].

*Candida* species, including *C. albicans*, *C. krusei*, and *C. glabrata*, are responsible for 8–10% of hospital-acquired (nosocomial) systemic mycoses, and are associated with mortality rates of up to 71%. The rising rate of fatal candidemia is compounded by emergent fluconazole- and voriconazole-resistant non-*Candida* strains [9]. The bis-oxazole natural products, bengazoles A (**2a**) and B (**2b**), also from *Jaspis* cf. *coriacea* [10,11], and their homologs C–G (**2c**–**g**) from an Australian *Jaspis* sp. [12], are potent antifungal agents (minimum inhibitory concentration, MIC, ~ 1 µg mL^–1^ [12]). 

The absolute stereochemistry of **1a** was determined by measuring the NMR anisotropy of the *S*- and *R*-mandelate esters of a related co-isolated lactone [10]. The absolute stereochemistry of **2a** was determined through comparisons of the CD spectra of the corresponding tetra *p*-bromobenzoate esters and synthetic models of defined configuration [12]. The first total syntheses of **1a [13]** (which confirmed the absolute configuration) and **2a [14,15]** were reported in 1992 and 1999, respectively, followed by several other bengamide syntheses; only two other syntheses of bengazoles have appeared [16,17]. The chemistry and the biology of bengamides and bengazoles were the subject of a recent comprehensive review [18].

A limited structure–activity study suggested that the two oxazole rings are required in **2a**–**g** for potent antifungal activity [19], as is the long fatty acyl chain. The saponification product of bengazoles A–G gave the pentaol **2h [10]** which was devoid of activity [12]), but few other substitutions are tolerated [19].

Bengamides―which co-occur with bengazoles in *Jaspis* sp.―lack antifungal activity against *C. albicans* yet remarkably, the anti-*Candida* activity of crude and partially purified *Jaspis* sp. extracts exceed the activity of purified bengazoles in the disk diffusion assay. For example, pure **2e** (MIC = 1 µM against *C. albicans* [12]) at 0.5 µg disk^−1^ induced a zone of inhibition of 9–10 mm against *C. albicans*, but a solvent-partitioned fraction containing a mixture of homologous bengazoles and bengamides created a zone of inhibition far larger (40 mm) at comparable loadings (unpublished results, see Figure S4). This observation suggested synergism in the multi-component mixture that may involve bengazole and bengamide interactions, or more complex activities that impaired fungal cell metabolism, leading to cellular collapse. 

**Figure 2:**
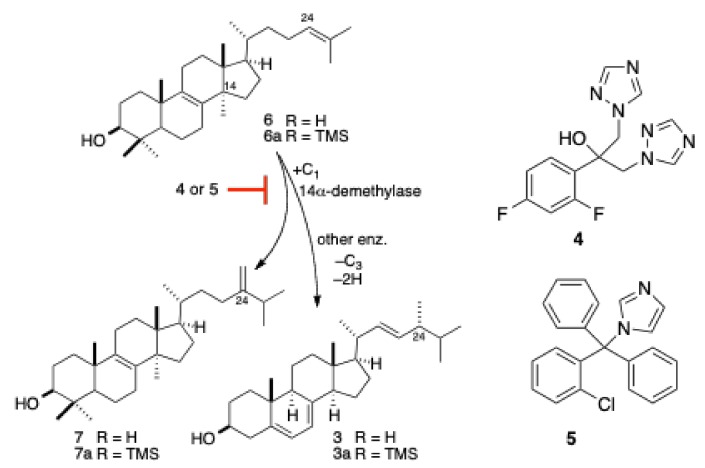
Oxidative remodeling in the bioconversion of lanosterol (**6**) to ergosterol (**3**) (abbreviated). Azole antifungal drugs, fluconazole (**4**) and clotrimazole (**5**), block lanosterol 14*α*-demethylase. Eburicol (**7**) is a shunt metabolite.

The precise molecular mechanism of action (MOA) of the bengazoles has not been determined, but we have shown that the antifungal activity of **2a** was suppressed in the presence of exogenous ergosterol (**3**) in a concentration-dependent manner [20], reminiscent of the MOA of polyene antifungal agents (e.g., amphotericin B [21,22]). The structure of **2a** bears some resemblance to clinically approved drugs—the so-called ”azole” antifungal agents (e.g., fluconazole, **4**, and clotrimazole, **5**, Figure 2). The latter embody two imidazole or pyrazole rings appended to a central carbinol, while **2** displays two 1,3-oxazole rings arrayed around an esterified carbinol. 

The structural similarities between antifungal azole drugs and **2** suggested that the two classes of compound might manifest the same MOA: namely, inhibition of lanosterol 14α-demethylase (We credit and thank Professor Yuzuru Shimizu (University of Rhode Island) for suggesting this hypothesis). The proposal is made more plausible by observations of inhibition of the growth of fungi by generic azoles as simple as imidazole itself [23], and the known mode of binding of azoles through coordination of the basic N to the Fe-heme core of 14α-demethylase [24]. Here, we report the results of the investigations of the MOA of bengazole natural products, including the antifungal synergism of bengazole A and bengamide A, and present the disproval of an azole-like suppression of ergosterol biosynthesis for the MOA of the former.

## 2. Results

Bengazoles are the rare, less-abundant, and less-studied components in extracts of *Jaspis* sp. Bengazoles are unstable: they are persistent in crude extracts, but upon purification, they undergo spontaneous degradation through autoxidation of the oxazole ring over short timescales. Crews et al. reported the co-isolation of compounds formed from the degradation of **2a**; their structures were expected products of photosensitized [4+2] additions of ^1^O_2_ to one of the 1,3-oxazole rings, followed by Wasserman-type fragmentation [25,26]. Remarkably, our type-sample extract of *Jaspis* sp. (90-026) collected from the Great Barrier Reef and stored in MeOH (–20 °C) for 25 years was found to retain antifungal activity. Remarkably, bengazoles, within crude extracts and prior to separation from other components, have much better stability and prolonged ”shelf life”. This useful phenomenon may be possibly attributed to photoprotection by pigments or antioxidant congeneric components in unrefined mixtures. Pure bengazole A (**2a**, 0.5 µg) gave a zone of inhibition of 9–10 mm [12]. The latter observation suggested the presence of intact bengazoles. In contrast, the bulk of the specimen had been extracted and purified to provide the major compounds, bengazoles A (**2a**) and B (**2b**), and minor homologs, C–G (**2c**–**g**), all of which subsequently decomposed [12].

### 2.1. Extraction–Isolation of Bengamides–Bengazoles

A portion of the MeOH supernatant from the type sample was separated by progressive solvent partition, and the CH_2_Cl_2_-soluble fraction was further purified by silica gel flash chromatography to yield a fraction containing a mixture of bengazoles (**2a**–**g**). Final purification by reversed-phase HPLC gave pure **2**, identified by MS and ^1^H NMR and comparison with literature values [10]. Purified **2a** (4 µg), determined with precision by microcryoprobe ^1^H NMR and quantitation using solvent ^13^C satellites (QSCS) [27,28], provided sufficient sample for limited quantitative antifungal assays. 

Ergosterol, the major sterol found in yeasts and other fungi, is a critical structural component that maintains the integrity of cellular membranes. Amphotericin B and related polyene antifungal agents exert their action by binding to ergosterol and inducing the formation of membrane pores that are permeable to K^+^ ions and other small-molecular-weight metabolites [29]. A common target exploited in the design of synthetic antifungal azoles is the inhibition of ergosterol biosynthesis [30], although recent efforts have been aimed at chitin-synthetase inhibitors [31]. Antifungal azoles inhibit the 14α-demethylase, an enzyme that is critical for oxidative remodeling of the common triterpene precursor lanosterol (**6**) (Figure 2) during ergosterol biosynthesis [32]. Although the structure of **2** is reminiscent of antifungal azoles, the ergosterol-dependent activity of the former suggests that the natural product targets ergosterol-lipid structured membranes by the formation of pores [21,29,30], although the possibility of a dual mode of action for **2** cannot be excluded. In order to test the latter hypothesis, the sterol composition of cultured *C. albicans* was monitored over time in the presence and absence of drugs that are known disruptors of ergosterol biosynthesis. 

### 2.2. Sterol Composition in C. albicans Co-Cultured with Azoles

**Figure 3:**
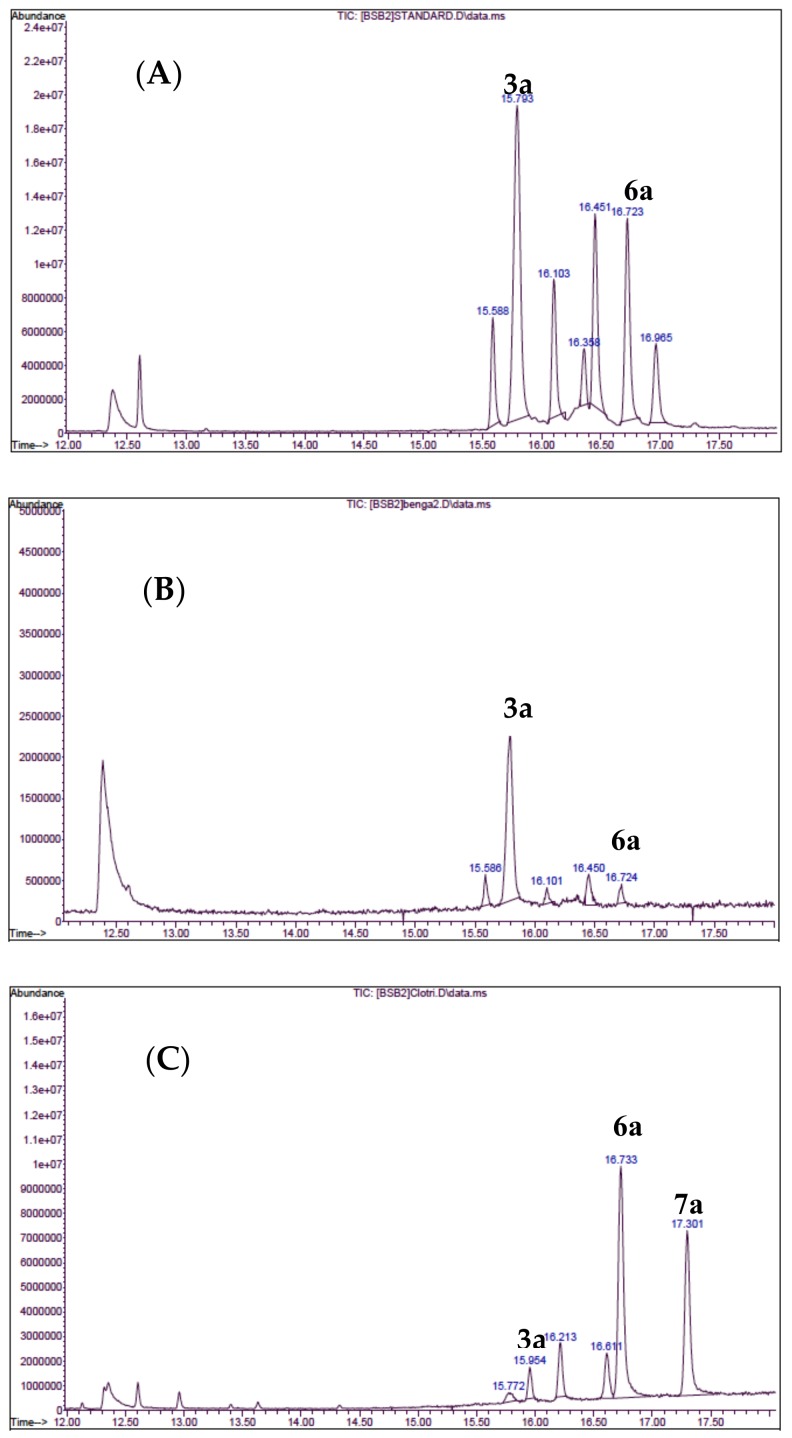
GCMS traces of *O*-TMS Sterols from *C. albicans* ATCC 14503 after co-incubation in the presence and absence of **2** and **5**. (**A**) *C. albicans* and media only. (**B**) C. *albicans* and **2**. (**C**) *C. albicans* and **5***. Key*: *O*-TMS ergosterol (**3a**), *m/z* 468, 378, 363, 337; *O*-TMS lanosterol (**6a**), *m/z* 498, 483, 393; *O*-TMS 24-methylenedihydrolanosterol (*O*-TMS eburicol, **7a**), *m/z 512, 497,* 407. For complete GCMS spectra, see Supporting Information.

Cultures of *C. albicans* ATCC 14503 were treated with the serially diluted bengazoles, and incubated overnight at 35 °C. The crude broths were centrifuged and growth inhibition was estimated on the basis of the cellular wet-weight of the pellets compared to the untreated control. Whole pellets were saponified (40% KOH in EtOH-H_2_O, 2 h, 95 °C), and the non-saponifiable fractions were recovered by extraction with ether, persilylated (*N*-(trimethylsilyl)imidazole), and the sterol composition was determined by GCMS (Figure 3) of the corresponding *O*-TMS ethers of ergosterol, lanosterol, and 24-methylenedihydrolanosterol (**3a**, **6a**, and **7a**, respectively, see Supporting Information). Growth inhibition was observed in the presence of **2**, but there was no change in the concentrations of **3** relative to the control (Figure 3b). To validate the method, azole **5** was tested under the same conditions. A clear decrease in **3** (Figure 3c) was seen along with the appearance of a new peak due to the *O*-TMS ether **7a** of the expected shunt metabolite, 24-methylenedihydrolanosterol (eburicol, **7**) [33,34].

### 2.3. Synergistic Antifungal Activity of Bengazole–Bengamide Mixtures

**Figure 4:**
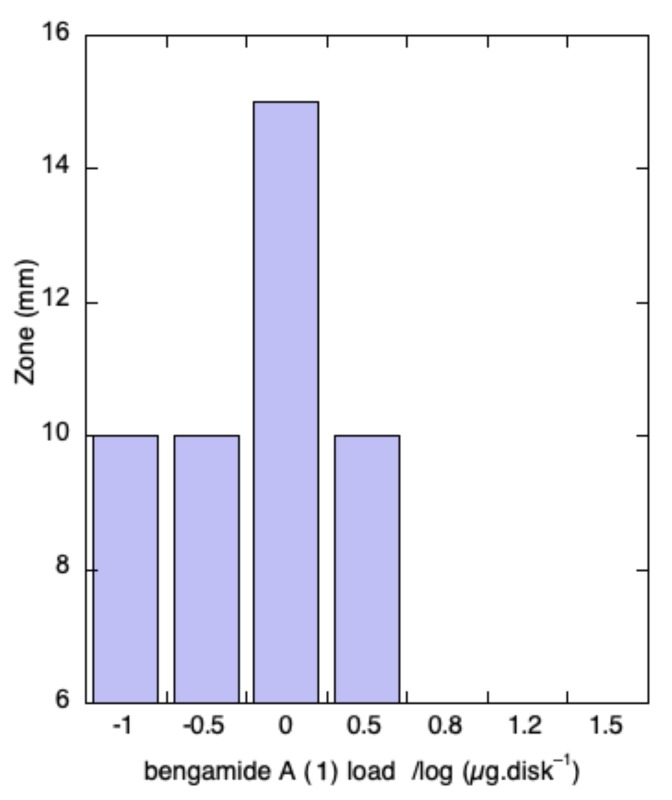
Antifungal disk diffusion assay *^a^* of bengazole *^b^*–bengamide mixtures *^b a^*. *^a^* A lawn of *Candida albicans* ATCC 14503 was spread onto Muller-Hinton (M-H) agar plates. Bengazoles (**2**, 0.5 µg disk^−1^) and bengamide A (**1**, variable loadings) were applied as solutions to the same 6 mm paper disk. The disks were dried, placed on M-H media agar plates, and incubated overnight at 35 °C. The diameter of the zone of inhibition was recorded (± 0.5 mm); *^b^* Under these conditions, bengazoles (**2**) alone (0.5 µg disk^−1^) gave a 10 mm zone of inhibition.

In order to investigate possible antifungal synergism, mixtures of bengazoles A–G (**2a**–**g**; hereafter referred to as **2** for brevity), at a constant loading (0.5 µg disk^−1^), and variable amounts of bengamide A (**1a**), were combined and tested in a disk diffusion assay (Figure 4). Surprisingly, at 200 µg disk^−1^ of **1a** (400:1 mass ratio of **1a**:**2**), complete inhibition of the antifungal activity of **2** was observed. When the relative concentration of **1a** was lowered, an unusual dose-dependent response was observed (Figure 4); **1a** inhibited antifungal activity of **2** at 15 µg disk^−1^ (30:1 ratio), but increased the zone of inhibition (~50%) at 1 µg of **2** (2:1 ratio of **1a** to **2**).

Further reductions in the ratio of **1a** to **2** showed no changes compared to the control. In contrast, the microbroth dilution assay using checkerboard [35] permutations of concentrations of **1a** and **2** did not reproduce the synergism observed with disk diffusion assays. As noted earlier, bengazoles are unstable. The time-dependent decomposition of **2** under the more prolonged incubation in the microbroth dilution assay or disk diffusion-related phenomena may account for the different responses under the two assay regimes. 

## 3. Discussion

Analysis of saponified cultures of *C. albicans* treated with clotrimazole (**5**) showed the appearance of eburicol (**7**)—a shunt metabolite sterol biosynthesis as expected—whereas cultures treated with bengazoles (**2**) did not. This implies that bengazole does not inhibit ergosterol biosynthesis in the same manner as **3** and other azole antifungal drugs which inhibit 14α-demethylase. In the latter case, the relatively higher ratio of lanosterol (**6**) to ergosterol (**3**) may suggest a buildup of the former—implying there is an inhibition of biosynthesis at an earlier stage―but absence of an internal standard limited conclusions on a more quantitative basis. We cannot exclude the inhibition of the biosynthesis of **3** by **2** at other steps in sterol biosynthesis (e.g., inhibition of squalene epoxidase [36,37]), however, testing this hypothesis will require an expanded, more quantitative experimental design. 

Synergistic activity in antibiotics is not uncommon [38,39], but is less-frequently reported for antifungal drugs [40]. We, and others, have noted synergism in antifungal microbroth dilution assays (MICs) of antifungal cyclic peptides, e.g. lobocyclamides from the cyanobacterium *Lyngbya majuscula* [41]. Moore and coworkers noted similar behavior in disk diffusion assays with the related peptides, laxaphycins A and B from the cyanobacterium *Hormothamnion enteromorphoides* (see Refs. [42,43] (interestingly, synergism was also observed for combinations of new laxaphycins in antiproliferative assays of cultured HCT-116 colon cancer cells [44]). Whether the synergistic fungal inhibition observed under microbroth dilution assays can be translated into improved efficacy in animal models of disseminated mycoses is a matter worthy of further study. 

It is worth commenting that many studies of naturally occurring marine products with antifungal properties [45], although reports of significant in vitro activity against cultured fungi, have lacked experimental proof that might illuminate details of MOA. These shortfalls may have side-lined potentially new mechanistic insights; yet speculative hypotheses abound. For example, the well-known antifungal activity of long-chain ”two-headed” aminoalkanols (e.g., oceanapiside [46]) found in sponges of the genera *Oceanapia, Rhizochalina*, *Leucetta,* and *Calyx* [47] were thought to exert antifungal activity by mimicking intermediates in the biosynthesis of sphingolipids and related long-chain bases [48], but evidence has mounted to support alternative models of the inhibition of fungal cell growth through actin-binding and the disruption of microfilaments [49]. Clearly, early identification of MOA is an asset in identifying viable leads from ”hits” in antifungal drug discovery campaigns.

Studies of antibiosis against bacteria that follow canonical MOAs are familiar (e.g., penicillins: inhibition of cell-wall biosynthesis; erythromycin: disruption of protein translation at rRNA [21]). In contrast, the characterization of antifungal MOAs has presented inordinately encumbered challenges, complicated by diploid pathogenic organisms that exhibit fewer genetic and metabolic differences from the host organism compared to bacteria. In the future, well-designed in vitro experiments that link read-outs of antifungal phenotypes with specific MOAs will be more desirable in screening-based discovery programs for natural products.

## 4. Materials and Methods

### 4.1. General Experimental Procedures

Inverse detected 2D NMR spectra were measured on a ECA (500 MHz) NMR spectrometer (Jeol, Peabody, MA, USA), equipped with a 5 mm ^1^H-{^13^C} probe, or an Avance III (600 MHz) NMR spectrometer (Bruker, Billerica, MA, USA), fitted with a 1.7 mm ^1^H-{^13^C} microcryoprobe. High-resolution ESITOF analyses were carried out on an Agilent 1200 HPLC coupled to an Agilent 6230 TOFMS (Agilent, Santa Clara, CA, USA), calibrated immediately before measurement against an ESL-L low concentration tuning mix (part number G1969-85000, Agilent Technologies). Low-resolution MS measurements were made on a Thermoelectron Surveyor UHPLC (Thermo Fisher, Waltham, MA, USA) coupled to an MSD single-quadrupole detector. HPLC was performed on an Agilent 1200 HPLC. Other General Experimental details can be found elsewhere [50].

### 4.2. Extraction and Purification of Bengazole A

An aliquot (5 mL) of the supernatant from *Jaspis* sp. (type sample 90-20-026), stored in MeOH (10 mL), was extracted with hexanes (5 mL × 2). Concentration of the hexane-soluble layer gave fraction A. The aqueous-MeOH layer was adjusted to 2:3 H_2_O:MeOH and extracted with CH_2_Cl_2_ (7 mL × 2) to yield, after removal of volatiles, fraction B (3.3 mg). Fraction B was separated by silica gel flash chromatography (stepped gradient, 2.5% MeOH increments in CH_2_Cl_2_ to 10:90 MeOH:CH_2_Cl_2_) followed by a 50:50 MeOH:CH_2_Cl_2_ wash, to yield five fractions (monitored by ^1^H NMR, 500 MHz). Fractions 4 and 5 were combined and purified by reversed-phase HPLC (Phenomenex Luna C_18_ column, 250 × 4.6 mm, linear gradient; initial conditions 25:75 H_2_O:CH_3_CN to 100% CH_3_CN over 17 min, 1 mL min^–1^ flow rate, UV 217 nm, and ELSD detection) to yield compound **2a** (4 µg, *t*_R_ = 11.8 min). The amount of **2** was quantitated by the solvent ^13^C-satellites (QSCS) method [28] using a ^1^H NMR microcryoprobe (600 MHz, CDCl_3_).

### 4.3. Quantification of Ergosterol from *C. albicans* ATCC 14503

A two-fold dilution series was prepared from Sabouraud-dextrose broth (SabDex, 2 mL) in each of 8 × 25 cm^2^ flasks. Overnight liquid cultures (10 µL) of *C. albicans* ATCC 14503 were added and incubated at 35 °C overnight with gentle shaking. The culture broths were centrifuged, washed (2 × PBS), and the wet pellet was weighed to estimated growth inhibition. The control pellet and one with 50% growth inhibition were saponified in 40% KOH solution (8:1 EtOH:water, 500 µL) at 95 °C for 2 hr. The non-saponifiable material was diluted with deionized H_2_O (2 mL) and vigorously extracted with diethyl ether (2×, vortex 30 s, followed by centrifugation, 5 min). The combined organic layers were dried under a stream of N_2_ and the residue dissolved in *n*-hexane (500 µL), then treated with *N*-(trimethylsilyl)imidazole (10 µL, Pierce). Samples were analyzed by GCMS (1 µL injection, 1.2 mL min^–1^ He, 70 °C for 2 min, ramp to 270 °C over 24 min, ramp to 300 °C at 35 min, EI detection). Sterols were identified (see Figure 3 and Supporting Information, S1–S3) by comparisons of peak *m/z* values, including fragmentation patterns, against entries from the NIST MS data library. Extracts of *C. albicans* liquid cultures, grown in the presence of growth inhibitory concentrations (50% reduction of cellular mass) of bengazole A or clotrimazole, were also analyzed by GCMS and compared with control cultures (see Figure 3).

### 4.4. Antifungal Disk Diffusion Assay

An overnight liquid culture of *C. albicans* ATCC 14503 was diluted 100-fold and spread on Mueller-Hinton agar plates. Aliquots of each compound were applied as solutions in MeOH onto sterile paper disks (6.0 mm). After five minutes, the disks were placed on the yeast-coated agar plates, and incubated overnight (~16 h) at 35 °C. Antifungal activity was estimated by the measurement of zones of inhibition (>6.0 mm ± 0.5 mm, Figure 4).

## 5. Conclusions

We reported the potential synergistic activity of bengamide A (**1**) and bengazoles. Remarkably, a 25-year old sample of *Jaspis* sp. collected from the Great Barrier Reef, Australia, retained potent antifungal activity, which guided the separation of **1** from **2** using an agar-based disk diffusion assay. Re-assay of the pure compounds confirmed that **1** was devoid of antifungal activity against *Candida albicans*, but mixtures of **1** and **2** displayed synergistic activity with optimum enhancement of the zone of inhibition at a ratio of 2:1. Although inhibition of fungal growth was observed in cultures of *C. albicans* in the presence of **2**, no suppression of ergosterol biosynthesis was detected, suggesting a MOA that differs from 14α-demethylase inhibition, common to the azole antifungal drugs. Further investigations into the MOA of potent antifungal bengazoles are in progress.

## Supplementary Materials

The following are available online at http://www.mdpi.com/1660-3397/17/2/102/s1. LCMS data of sterol *O*-TMS ethers and first record of ”synergism”.
