# Supplementary material for: Synergistic Anti-Candida Activity of Bengazole A in the Presence of Bengamide A
Source: Mar Drugs. 2019 Feb 7;17(2):102. doi: 10.3390/md17020102 (PMC6410253; doi:10.3390/md17020102)
Supplement: Supplementary file 1 [file marinedrugs-17-00102-s001.pdf]

Supporting Information

Synergistic Anti-*Candida* Activity of Bengazole A in the Presence of Bengamide A

Matthew T. Jamison[†] and Tadeusz F. Molinski*[,†,§]

[†]*Department of Chemistry and Biochemistry and* [§]*Skaggs School of Pharmacy and Pharmaceutical Sciences, University of California, San Diego, 9500 Gilman Dr. MC-0358, La Jolla, California 92093-0358.*

Page	Title	Content
S1	Figure S1	LCMS of TMS-sterols from *Candida albicans*, Incubated with Media, Only.
S2	Figure S2	LCMS of TMS-sterols from *C. albicans*, Incubated with Bengazole A (**2**).
S3	Figure S3	LCMS of TMS-sterols from *C. albicans*, Incubated with Clotrimazole (**5**).
S4	Figure S4	Notebook page, see Reference #15.

```
File        :D:\GCMS_DATA\2015\100715-Matthew-Sterol\BSB\STANDARD.D
Operator    : [BSB2]Matt
Acquired    :  7 Oct 2015  16:36     using AcqMethod STEROL-TMS.M
Instrument :    GCMS
Sample Name: Standard
Misc Info   :
Vial Number: 2
```

Figure S1. LCMS of TMS-sterols from *Candida albicans*, Incubated with Media, Only.

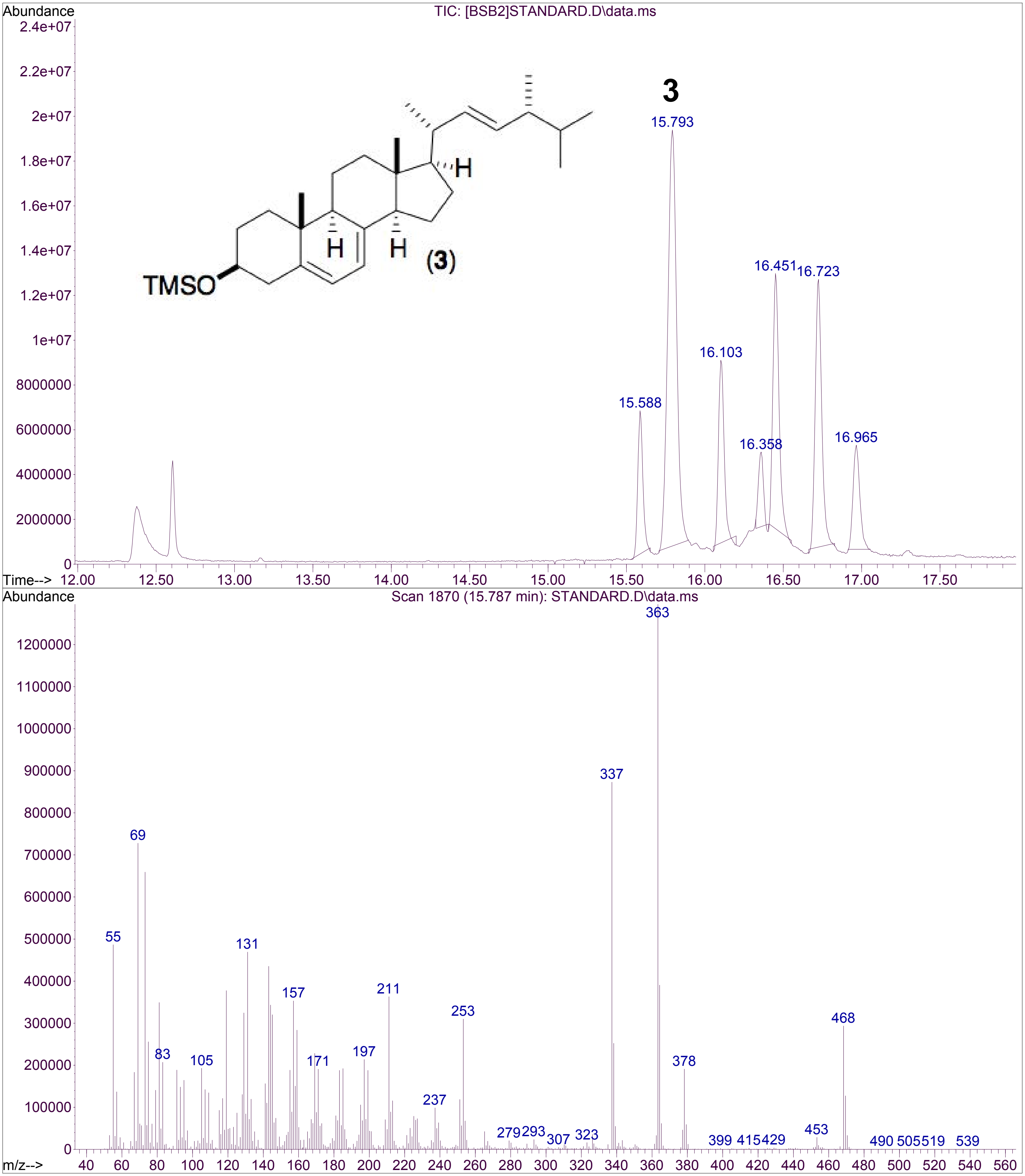

```
File        :D:\GCMS_DATA\2015\100715-Matthew-Sterol\BSB\benga2.D
Operator    : [BSB2]Matt
Acquired    :  7 Oct 2015  17:06     using AcqMethod STEROL-TMS.M
Instrument :     GCMS
Sample Name: benga2
Misc Info   :
Vial Number: 3
```

Figure S2. LCMS of TMS-sterols from *Candida albicans*, Incubated with Bengazole A (**2**)

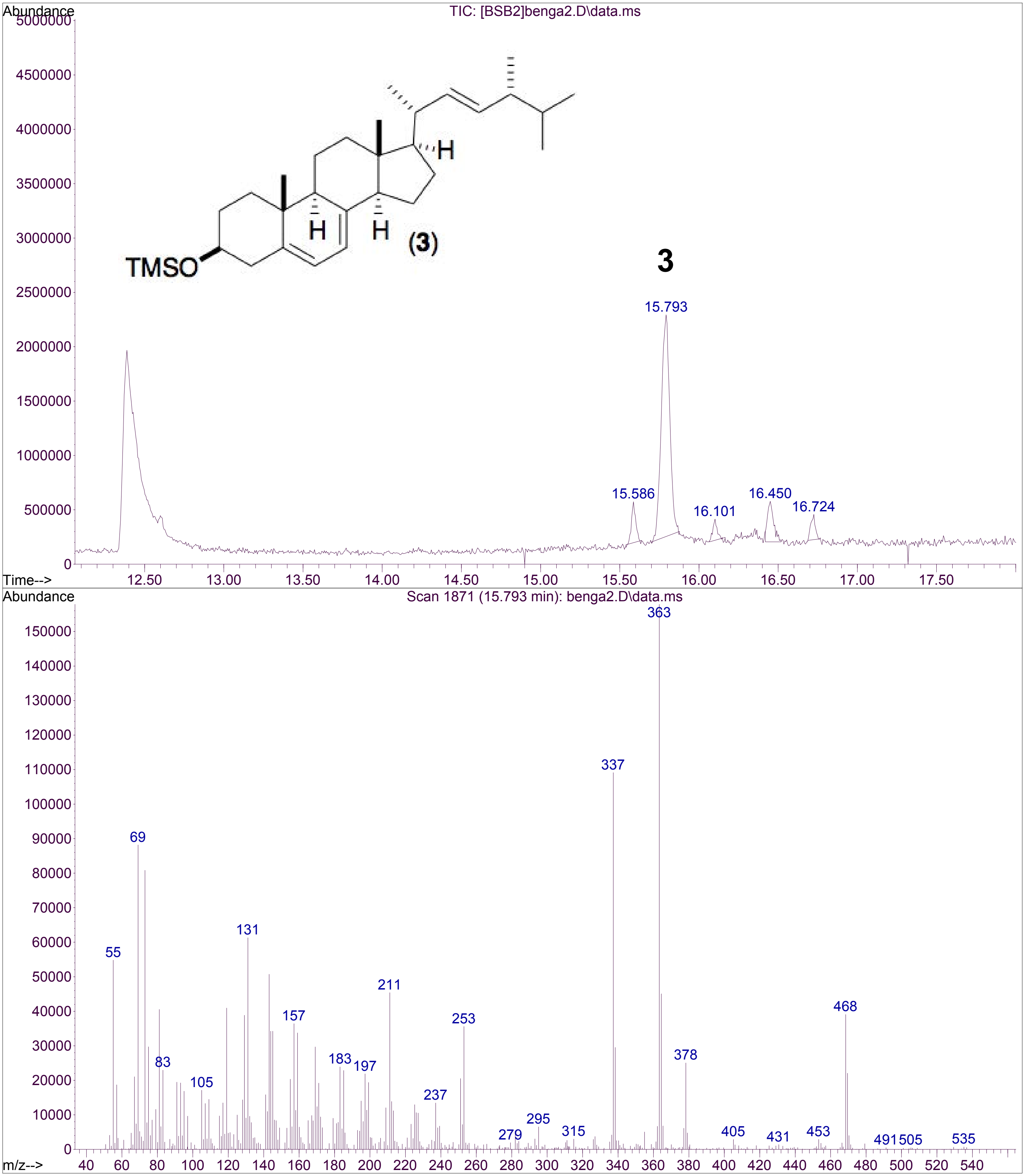

```
File        :D:\GCMS_DATA\2015\091715-Matthew-Sterol\BSB\Clotri.D
Operator    : [BSB2]Biswa
Acquired    : 17 Sep 2015  17:50     using AcqMethod STEROL-TMS.M
Instrument  :    GCMS
Sample Name: Clotri
Misc Info   :
Vial Number: 4
```

Figure S3. LCMS of TMS-sterols from *Candida albicans,* Incubated with Clotrimazole(**5**)

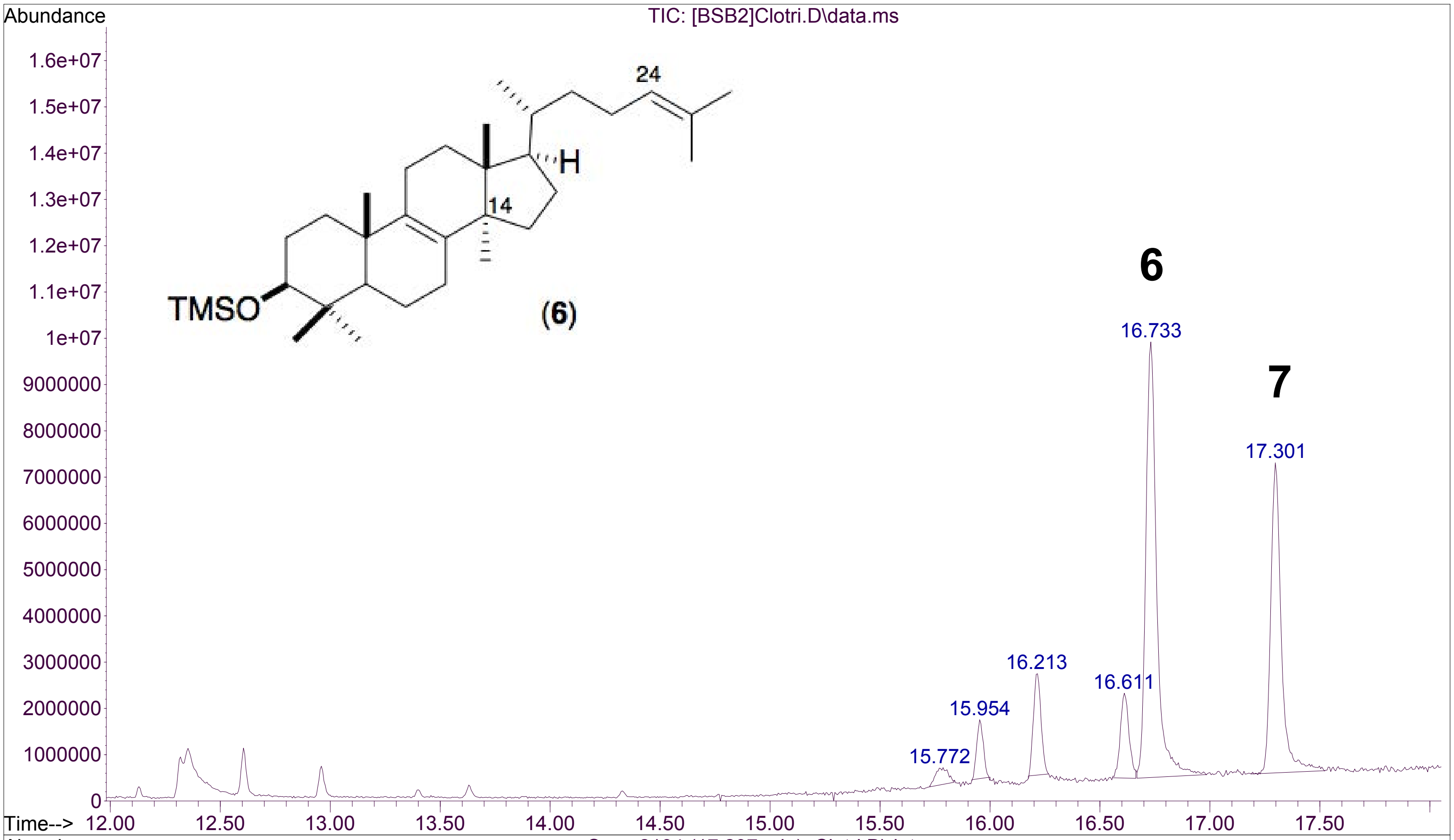

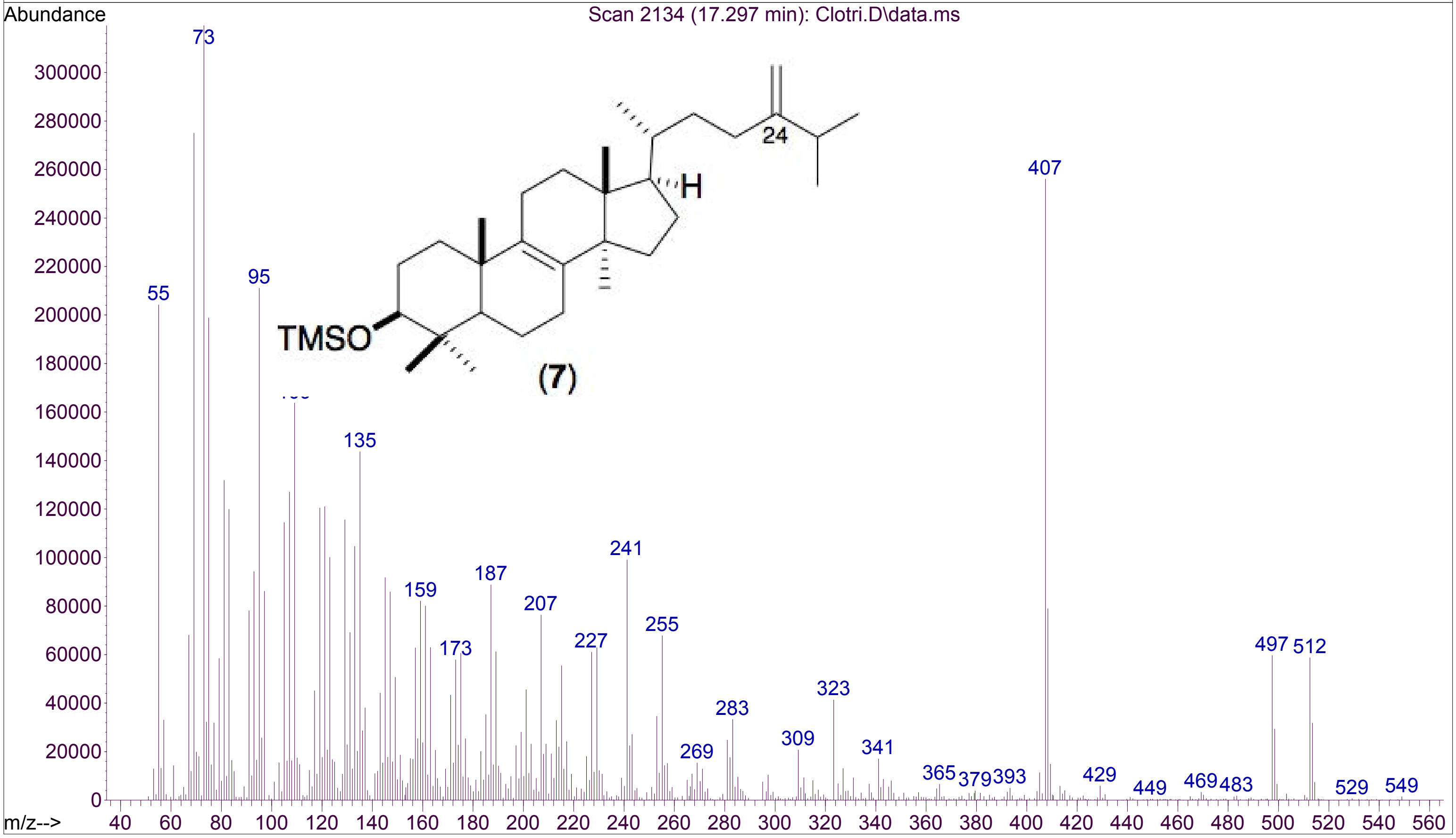

BENGAZOLES
BENGAMIDES
[illegible]0-04-026

Figure S4: See footnote #2

90-04-026

06/11/90 0036

Sponge :- Bright orange, encrusting, small. Grown with conglomerated coral rubble, shells (bivalves), etc.

Location :- Collected Norman Reef, -10 m, 05/10/90. Great Barrier Reef, Australia.

Identification :- 02192 Jaspis sp. (MK Harper, Scripps)

Activity :- C. albicans +++++ ?

Extraction :- Freeze dried entire sample. (48h). Dry material (13.50 g) soaked in methanol (24h). Solvent extract filtered off and fresh methanol added to sponge 06/12/90
Extracted residue of sponge =

1st Methanol extract :- Partitioned according to Kupchan scheme as follows. Total volume of MeOH extract = 230 mL.

90-04-026-1 :- Added 10% v/v H_2O (23 mL), extracted once with equal volume of n-hexane, followed by second extraction with 1/3 volume (70 mL). Combined hexane (upper) layers, dried (Na_2SO_4) and evaporated to give yellow brown oil (117.7 mg)

90-04-026-2 :- Added further 23 mL of H_2O (total 20% v/v) to lower phase and extracted, ~~as above with~~ but with CCl_4 as described above. Obtained green yellow oil (92.8 mg.)

90-04-026-3 :- Added 46 mL H_2O (total 40% v/v H_2O) and extracted with $CHCl_3$ as described above to obtain yellow/green oil (137.8 mg) Soluble in MeOH / insoluble in $CHCl_3$.

Aqueous methanol phase was lyophilized (1.10 g.) pale green white solid
90-04-026-4.

Testing Against Candida albicans @ ~~[illegible]~~ disk.

90-04-026-	1	-2	-3	-4	
	0	>40 mm*	~20 mm*	0	⇐ 15 µg /disk
	0	9 mm	9 mm	0	⇐ 1.5 µg /disk

* Irregular
